# Oxygen- and pH-Dependent Photophysics of Fluorinated Fluorescein Derivatives: Non-Symmetrical vs. Symmetrical Fluorination

**DOI:** 10.3390/s20185172

**Published:** 2020-09-10

**Authors:** Ciaran K. McLoughlin, Eleni Kotroni, Mikkel Bregnhøj, Georgios Rotas, Georgios C. Vougioukalakis, Peter R. Ogilby

**Affiliations:** 1 Department of Chemistry, Aarhus University, DK-8000 Aarhus, Denmark; ciaran_mcl@hotmail.com (C.K.M.); mibr@chem.au.dk (M.B.); 2 Department of Chemistry, National and Kapodistrian University of Athens, 15771 Athens, Greece; helen.kotroni@gmail.com (E.K.); rotasgiorgos@hotmail.com (G.R.)

**Keywords:** singlet oxygen, fluorescent probe, photobleaching

## Abstract

Fluorescein, and derivatives of fluorescein, are often used as fluorescent probes and sensors. In systems where pH is a variable, protonation/deprotonation of the molecule can influence the pertinent photophysics. Fluorination of the xanthene moiety can alter the molecule’s p*K*_a_ such as to render a probe whose photophysics remains invariant over a wide pH range. Di-fluorination is often sufficient to accomplish this goal, as has been demonstrated with compounds such as Oregon Green in which the xanthene moiety is symmetrically difluorinated. In this work, we synthesized a non-symmetrical difluorinated analog of Oregon Green which we call Athens Green. We ascertained that the photophysics and photochemistry of Athens Green, including the oxygen-dependent photophysics that results in the sensitized production of singlet oxygen, O_2_(a^1^Δ_g_), can differ appreciably from the photophysics of Oregon Green. Our data indicate that Athens Green will be a more benign fluorescent probe in systems that involve the production and removal of O_2_(a^1^Δ_g_). These results expand the available options in the toolbox of fluorescein-based fluorophores.

## 1. Introduction

Fluorescein, and many of its derivatives, have long been recognized as useful fluorescent probes [1]. This includes their use in a variety of biological imaging experiments [2]. In this regard, the light emitted is at a readily detected wavelength (~500 nm), the quantum yields of emission are large (>0.9), the molecules are generally photostable, and they do not sensitize the production of singlet molecular oxygen, O_2_(a^1^Δ_g_), in appreciable yield. Although mechanisms of fluorophore photobleaching are often complicated and depend on the molecule’s structure and whether or not molecular oxygen is present in the system, it is acknowledged that O_2_(a^1^Δ_g_) can be a key intermediate in this regard [3,4,5,6,7]. Thus, a low yield of sensitized O_2_(a^1^Δ_g_) production not only helps mitigate photobleaching, but it also helps to minimize the extent to which the fluorescein perturbs the system in which it is used as a probe by minimizing the O_2_(a^1^Δ_g_)-mediated oxidative degradation of other molecules.

**Figure 1:**
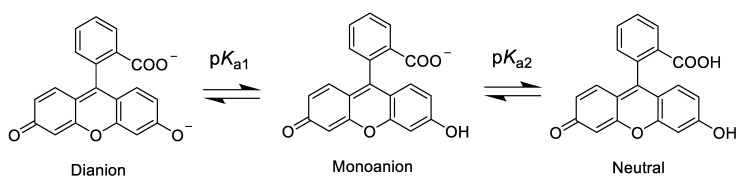
Scheme illustrating the equilibrium between the neutral, monoanionic, and dianionic forms of fluorescein.

Xanthene-based molecules such as fluorescein have a feature that influences their use as a fluorescent probe: pH-dependent protonation/deprotonation alters properties of the molecule. In this way, for example, the extent of aggregation and intermolecular binding [8,9], the magnitude of the fluorescence quantum yield [10,11,12,13], and the yield of photosensitized O_2_(a^1^Δ_g_) production [12,14] all depend on whether the molecule is in its dianion, monoanion, or neutral form (Figure 1). Although more complicated schemes can be presented to illustrate the pH-dependent structures of fluoresceins [15,16], the equilibria shown in Figure 1 are sufficient for our current discussion.

It has long been recognized that fluorinating the xanthene ring in fluorescein and its derivatives is one way to change the equilibrium constants shown in Figure 1, particularly that between the dianion and monoanion upon which much of the photophysics in common aqueous solutions depends [12,14,15,16,17,18]. Specifically, upon replacing the xanthene hydrogen atoms with more electronegative fluorine atoms, one stabilizes the dianion yielding a smaller p*K*_a1_ value. In this way, one can use the fluorescein derivative over a wider pH range without a protonation-dependent change in photophysical properties. Fluorination of a chromophore/fluorophore can also result in a molecule that is more stable to photooxidative degradation, partly due to a decrease in the yield of photosensitized O_2_(a^1^Δ_g_) production and partly to a decreased reactivity with electrophiles such as O_2_(a^1^Δ_g_) [7,12,14,19].

**Figure 2:**
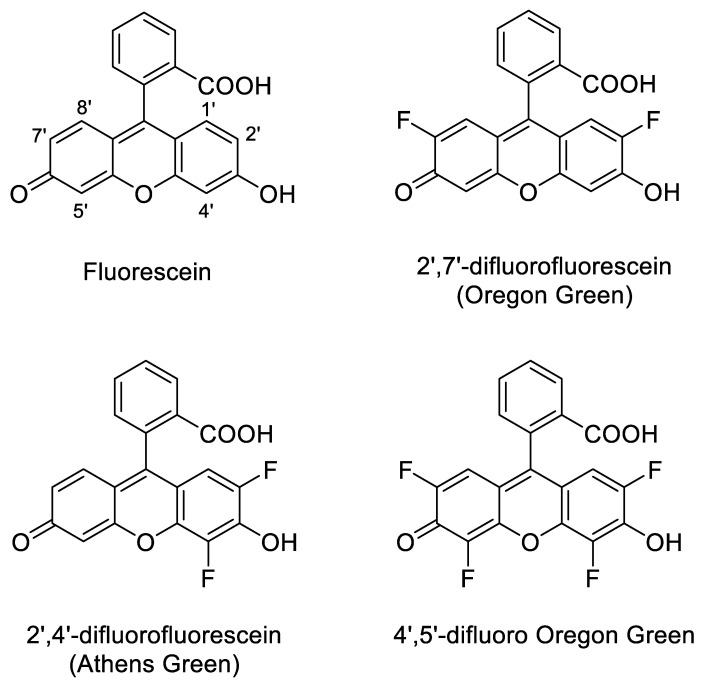
Chemical structures of the compounds investigated in this study. The standard numbering system for fluorescein is shown in the top left.

Although a variety of fluorinated fluorescein derivatives have been synthesized and studied over the years [12,14,15,16,17,18], the molecules produced have almost exclusively had the fluorine atoms symmetrically disposed on the xanthene moiety of the molecule. Representative examples include Oregon Green and 4′,5′-difluoro Oregon Green (Figure 2). To our knowledge, there is only one report on a non-symmetrical 2′,4′-difluorofluorescein, and this work focused on synthesis rather than on photophysical studies [20].

Most importantly, and again to our knowledge, studies that compare the photophysical properties of symmetrical and non-symmetrical fluorinated fluorescein derivatives have yet to be performed. The potential for appreciable differences in these properties, due to the anticipated changes in electron density distribution, is sufficient justification for a study of selected photophysical parameters. However, even if photophysical differences are small, the synthetic procedures used to prepare a given fluorescein derivative may be more easily realized for the non-symmetrical isomer and thus provides additional justification for this work. For example, it is acknowledged that the synthesis of the tetra-fluorinated fluorescein derivative called Aarhus Sensor Green is challenging [14], and it would be beneficial if other more readily prepared fluorinated derivatives have photophysical properties that are just as acceptable.

From a general synthetic point of view, there are only a few reports on the preparation of non-symmetrically functionalized fluorescein derivatives. The latter has generally been achieved via two consecutive Friedel–Crafts acylation reactions with substituted phenols [20,21]. The main problem in these syntheses is the undesired formation of symmetrical derivatives [22]. As such, in itself, this is an issue that deserves attention.

**Figure 3:**
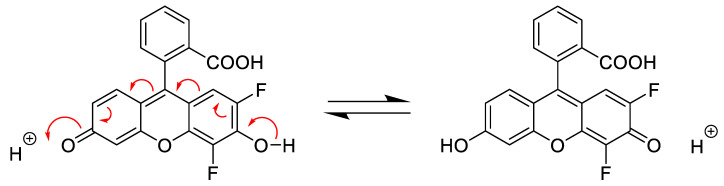
Tautomerization that shows the effective equivalence of the 2′,4′- and 5′,7′-difluorofluoresceines.

For the present work, we provide a study on the synthesis of the non-symmetrical 2′,4′-difluorofluorescein. Moreover, we compare selected photophysical properties of this non-symmetrical derivative to those obtained from the symmetrical 2′,7′-isomer (i.e., Oregon Green), the tetra-fluorinated fluorescein (i.e., 4′,5′-difluoro Oregon Green), and fluorescein itself (Figure 2). Given the tautomerization shown in Figure 3 and the standard numbering system shown in Figure 2, the 2′,4′-difluorofluorescein could also be identified as 5′,7′-difluorofluorescein.

It has become a convention when making new fluorescein derivatives to name the compound according to the place in which it was first made. Alongside the proper IUPAC nomenclature, this reversion to “common” nomenclature certainly facilitates conversations about the respective compounds. Thus, we now have Tokyo Green [23], Oregon Green [17], Pennsylvania Green [18], Singapore Green [24], Granada Green [25], and Aarhus Green [12], for example. In this spirit, we refer to our new 2′,4′-difluorofluorescein as Athens Green (Figure 2).

## 2. Results and Discussion

### 2.1. Synthesis of Athens Green

**Scheme 1:**
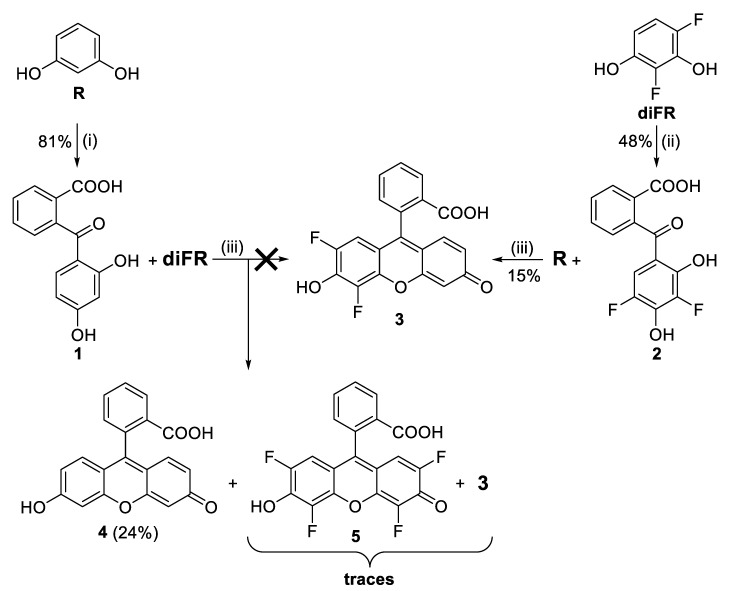
Synthetic approaches used to produce Athens Green, **3**. Reagents and conditions: (i) **PA**, AlCl_3_, CH_3_NO_2_, room temperature, (ii) **PA**, AlCl_3_, CH_3_NO_2_, 120 °C, (iii) a) CH_3_SO_3_H, b) Et_3_N. The yields of each reaction are shown as percentages.

As shown in Scheme 1, we approached the synthesis of the non-symmetric difluoro derivative through two independent stepwise Friedel–Crafts reactions of resorcinol (**R**), 2,4-difluororesorcinol (**diFR**), and phthalic anhydride (**PA**). We prepared **diFR** in four steps from 2,3,4,5-tetrafluoronitrobenzene [14].

In this way, the fluorine-free ketone **1** was prepared using the standard AlCl_3_-mediated procedure. To add the fluorine atoms, **1** was then subjected to a second Friedel-Crafts reaction with **diFR**, this time using methanesulfonic acid both as the catalyst and the solvent [20,22]. Published reports indicate that similar reactions were performed at low temperature to avoid the formation of symmetric fluoresceins stemming from competing Friedel-Crafts reactions [20,22]. In our hands, the reaction between **1** and **diFR** afforded non-fluorinated fluorescein **4** as the main product, together with other unidentified products and only traces of **3** and **5** (Scheme 1). Performing the reaction at 0 °C, or ambient temperature, or by using excess **diFR**, did not change the outcome. In a different approach, we prepared ketone **2** using the Friedel-Crafts reaction between **PA** and **diFR**. The low nucleophilicity of the latter required heating to 120 °C to obtain **2** in moderate yield. Reaction with **R** then afforded the desired non-symmetrical difluoride **3** in low but reproducible yield.

**Scheme 2:**
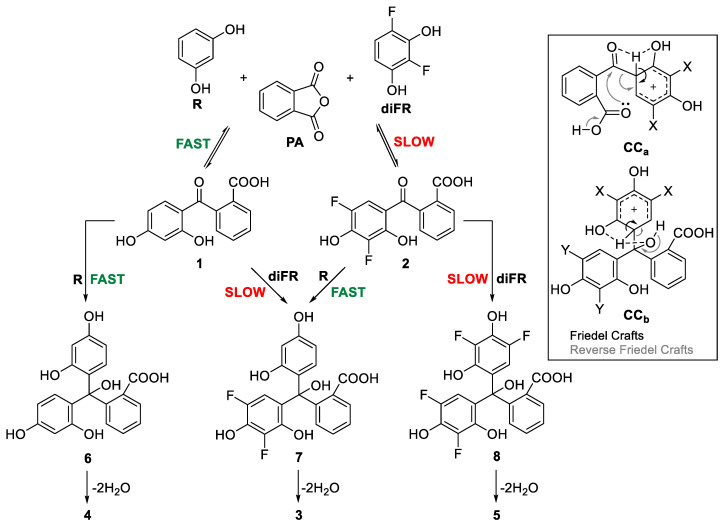
Proposed mechanistic representation of the formation of fluoresceins **3**, **4,** and **5** (i.e., Athens Green, fluorescein, and 4′,5′-difluoro Oregon Green, respectively).

We propose the mechanism shown in Scheme 2, which can rationalize why the reaction of ketone **1** with **diFR** principally produces fluorescein **4**, while the reaction of ketone **2** and **R** affords mainly difluorinated fluorescein **3** (i.e., Athens Green). It is first important to recognize that, because we work under acidic conditions, we should consider all reactions shown in Scheme 2 as equilibria (i.e., Friedel-Crafts and reverse Friedel-Crafts reactions). Thus, for example, in the reaction of ketone **1** and **diFR**, formation of the Friedel-Crafts product **7** will compete with the formation of **R** and **PA** via a reverse Friedel-Crafts reaction. In both the Friedel-Crafts and reverse Friedel-Crafts reactions, similar arenium carbocation intermediates are formed (**CC_a_** and **CC_b_** in Scheme 2), either from the nucleophilic attack of the aromatic ring on a carbonyl carbon or a proton. These carbocations may be deprotonated (Friedel-Crafts) or decarbonylated (reverse Friedel-Crafts). When substituted with the electronegative fluorine atoms (X = F in Scheme 2), the carbocations are less stable. As such, if arenium carbocation formation is the rate-determining step of both the Friedel-Crafts and the reverse Friedel-Crafts reactions, as expected for a typical electrophilic aromatic substitution reaction [26], any reaction involving **diFR**, either as reactant (Friedel-Crafts) or product (reverse Friedel-Crafts) should be less favorable and, thus, slower. Therefore, the reaction of **2** with **R** is the best way to obtain the non-symmetrical difluoride **3** (i.e., Athens Green).

### 2.2. Photophysics and Photochemistry

Photophysical measurements were performed using both D_2_O- and H_2_O-based solutions. The rationale for this is the desire to perform selected oxygen-dependent studies in D_2_O where O_2_(a^1^Δ_g_) has a much longer lifetime than in H_2_O [27,28].

#### 2.2.1. Absorption and Fluorescence Spectra

**Figure 4:**
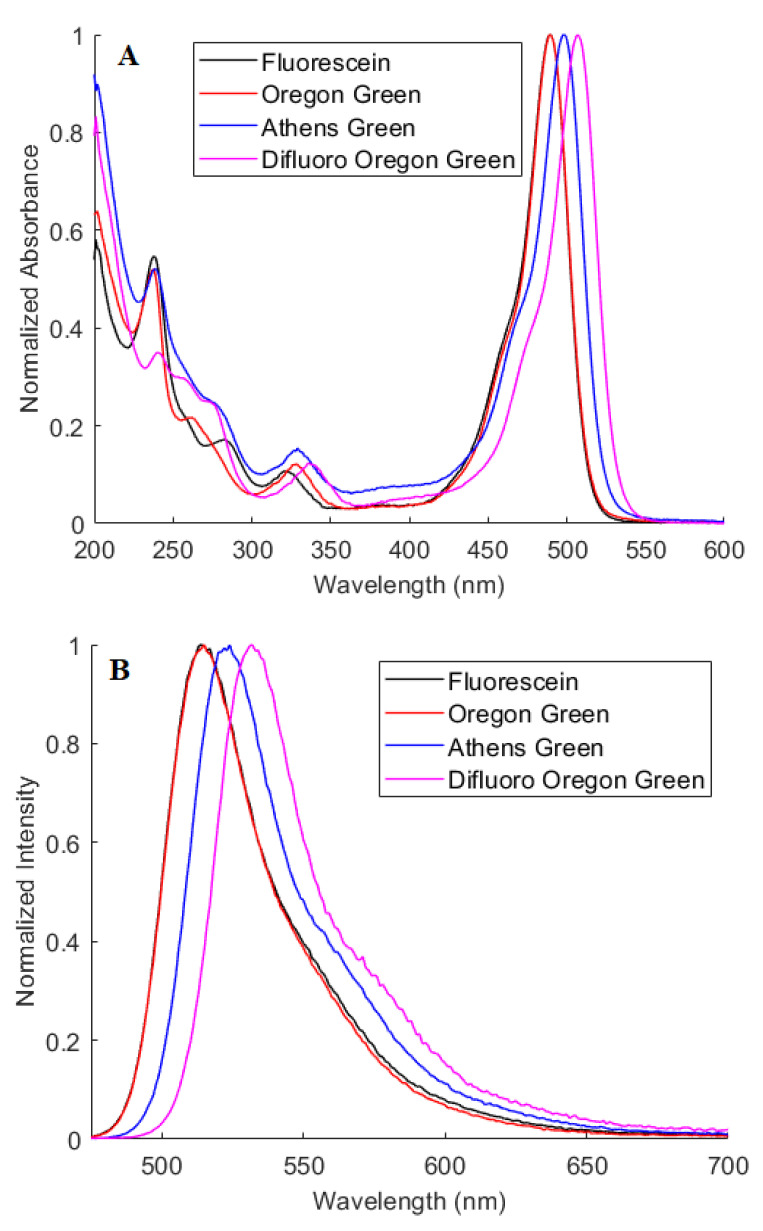
Normalized absorption (**A**) and fluorescence (**B**) spectra of the four compounds dissolved in D_2_O-PBS (pD = 7.8). Spectra recorded from H_2_O-PBS solutions are identical. Note that the spectra for fluorescein and Oregon Green are similar (black and red lines, respectively).

The absorption and fluorescence spectra of our four compounds dissolved in a phosphate-buffered D_2_O solution (pD = 7.8 = pH + 0.4 [29,30]) are shown in Figure 4. As outlined further below, the spectra at this pD mostly reflects the properties of the dianion of each compound.

Our results show that fluorination generally results in a bathochromic shift of the band maximum in both the absorption and emission spectra (Figure 4 and Table 1). This observation is consistent with data published on related compounds [20]. Most interestingly, however, the spectra of the symmetrical 2′,7′-difluoro compound (i.e., Oregon Green) are not appreciably different from those of fluorine-free fluorescein, whereas the spectra of the non-symmetrical 2′,4′-difluoro derivative (i.e., Athens Green) are noticeably red-shifted by ~10 nm. Fluorination in all four positions to form 4′,5′-difluoro Oregon Green results in a further red-shift of ~10 nm.

**Figure 5:**
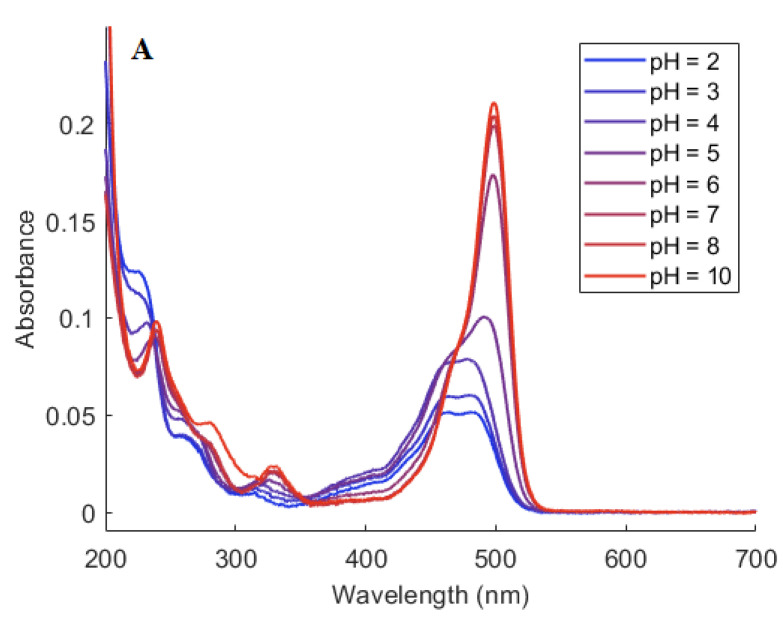

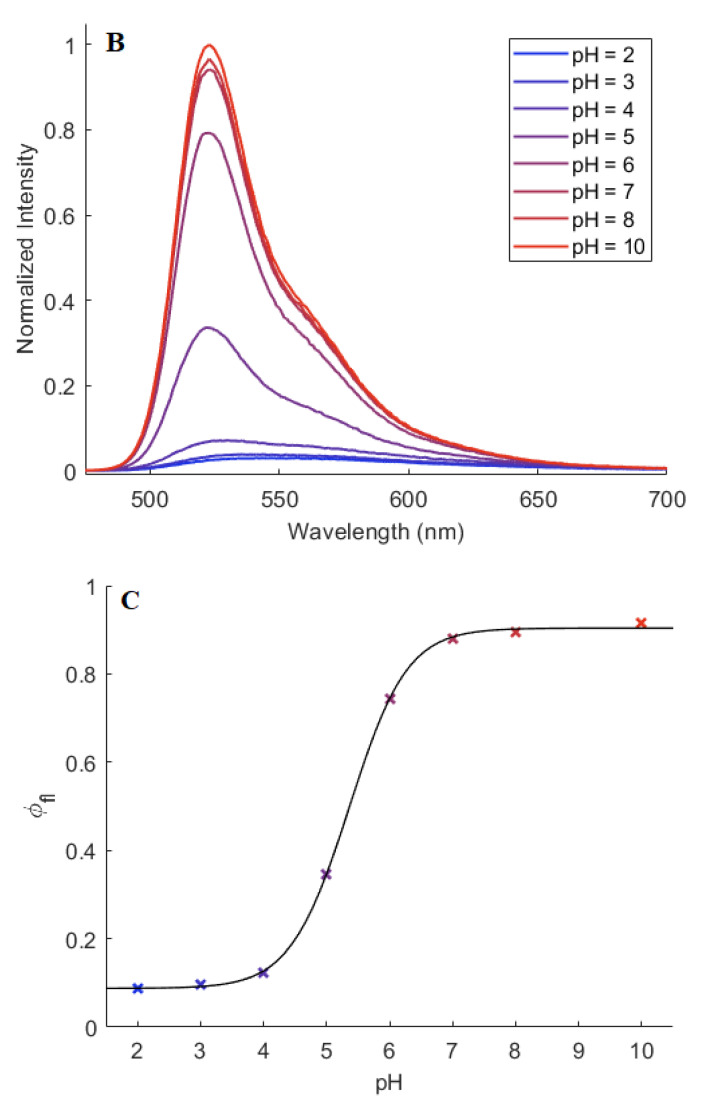
(**A**) Absorption spectra, (**B**) fluorescence spectra, and (**C**) fluorescence quantum yield, *φ*_fl_, of Athens Green in H_2_O recorded as a function of pH. For the latter, errors on a given point are approximately 5% of the *φ*_fl_ magnitude shown. For the fluorescence data, the excitation wavelength was 470 nm, and the spectra in panel B were normalized for pH-dependent changes in the sample absorbance at 470 nm. The *φ*_fl_ titration curve yields a p*K*_a1_ value of 5.3 ± 0.2. The second p*K*_a_ value is not evident from these data likely because of similar fluorescence and absorption properties of the monoanionic and neutral forms of Athens Green.

As expected, changing the pH/pD of the solution causes pronounced spectral and intensity changes in the absorption and emission spectra of all compounds. This is illustrated in Figure 5A for Athens Green where acidification results in a ~50 nm blue shift of the principal absorption band in the visible region of the spectrum. Although a corresponding spectral shift is not observed in the fluorescence spectrum of Athens Green, acidification results in an appreciable decrease in the fluorescence intensity (Figure 5B).

#### 2.2.2. Lifetimes of the Fluorescent State

Time-resolved fluorescence experiments were used to quantify excited singlet state lifetimes as a function of the oxygen concentration in our aqueous solutions (Table 2). In general, the values of ~4–5 ns thus obtained are consistent with what is expected based on data from related fluoresceins [31,32]. For all compounds, the lifetimes are shorter (Table 2) and the fluorescence quantum yields are smaller (Table 1) for molecules dissolved in H_2_O as opposed to D_2_O. This phenomenon has been independently observed for fluorescein and was interpreted to indicate that the O–H bond in the solvent plays an important role as an energy acceptor in the process of fluorescein internal conversion [31]. A similar argument has long been presented for an analogous H_2_O/D_2_O solvent isotope effect on the lifetime of O_2_(a^1^Δ_g_) [28,33].

There is no apparent correlation between the lifetime and the extent of xanthene fluorination. However, for all molecules, the lifetime appears to systematically get shorter as the oxygen concentration in the sample is increased (Table 2). Given the magnitude of the changes observed and errors of our measurements, however, we are hesitant to use this observation to extract a rate constant for the quenching of the excited singlet state by oxygen. Nevertheless, given that the concentration of dissolved oxygen in water is low [34], and that we are seeing a systematic effect of oxygen on lifetimes with a magnitude of 4–5 ns, implies that this rate constant is at or near the diffusion-controlled limit of ~10^10^ s^−1^ M^−1^. This is the conventional expectation for the quenching of a fluorescent state by oxygen [35,36].

#### 2.2.3. Fluorescence Quantum Yields

The decrease in the fluorescence intensity of Athens Green upon acidification (Figure 5B) reflects a corresponding decrease in the fluorescence quantum yield (*φ*_fl_). The latter is an expected observation based on data from other fluorescein derivatives in which protonation of the highly fluorescent dianion yields the weakly fluorescent monoanion [12,20]. The resultant titration curve (Figure 5C) yields a p*K*_a1_ value of 5.3 ± 0.2 for Athens Green in H_2_O. This p*K*_a_ value for the non-symmetrical difluoro derivative is slightly larger than those reported for the symmetrical difluoro derivative Oregon Green (4.8 and 5.1) [15,17]. This difference in p*K*_a_ values is consistent with what we independently recorded in our O_2_(a^1^Δ_g_) experiments (vide infra).

For data recorded under alkaline conditions, our fluorescence quantum yield of ~0.9 for Athens Green (Table 1, Figure 5C) is larger than the quantum yield (*φ*_fl_ = 0.63) reported for the related non-symmetrical difluorinated compound in which the pendant aryl group has two carboxyl groups instead of one [20]. Moreover, we obtain a p*K*a value of 5.3 for Athens Green, whereas a p*K*a value of 5.6 was obtained for the dicarboxylated analog [20].

These data recorded from different difluoro derivatives indicate that presumed subtle structural changes, including those on moieties removed from the xanthene core, can have appreciable photophysical consequences.

#### 2.2.4. Sensitized Production of O_2_(a^1^Δ_g_)

Based on the data presented above, we expect only small yields of fluorescein-sensitized O_2_(a^1^Δ_g_) production at pH > ~7. Specifically, we observe comparatively large quantum yields of fluorescence (Table 1) and that oxygen does not effectively quench the excited singlet state to promote intersystem crossing (i.e., the longer-lived triplet state would be a suitable O_2_(a^1^Δ_g_) precursor). However, upon acidification, both monoanionic and neutral fluoresceins can sensitize the production of O_2_(a^1^Δ_g_) in greater yield [12,14,37]. Moreover, even if produced in only small amounts, O_2_(a^1^Δ_g_) can lead to the photooxidative bleaching of the fluorophore [6,7] which, in turn, can result in the production of an efficient O_2_(a^1^Δ_g_) sensitizer [37,38]. It is thus incumbent upon us to quantify pH-dependent yields of O_2_(a^1^Δ_g_) sensitized by these fluorescein derivatives.

The ability of our compounds to sensitize the production of O_2_(a^1^Δ_g_) was quantified by monitoring the characteristic 1275 nm phosphorescence of O_2_(a^1^Δ_g_) in time-resolved experiments. Because the lifetime of O_2_(a^1^Δ_g_), *τ*_Δ_, is longer in D_2_O (67 μs) than in H_2_O (3.5 μs) [28], it is an advantage to perform these experiments in D_2_O solutions where the quantum efficiency of O_2_(a^1^Δ_g_) phosphorescence is correspondingly greater. Moreover, with a longer O_2_(a^1^Δ_g_) lifetime, it becomes easier to discriminate between O_2_(a^1^Δ_g_) removal and O_2_(a^1^Δ_g_) production; the latter generally occurs with time constants of ~3–5 μs in aqueous solutions due to the slow rate of sensitizer deactivation by the comparatively low concentration of oxygen [28,39].

**Figure 6:**
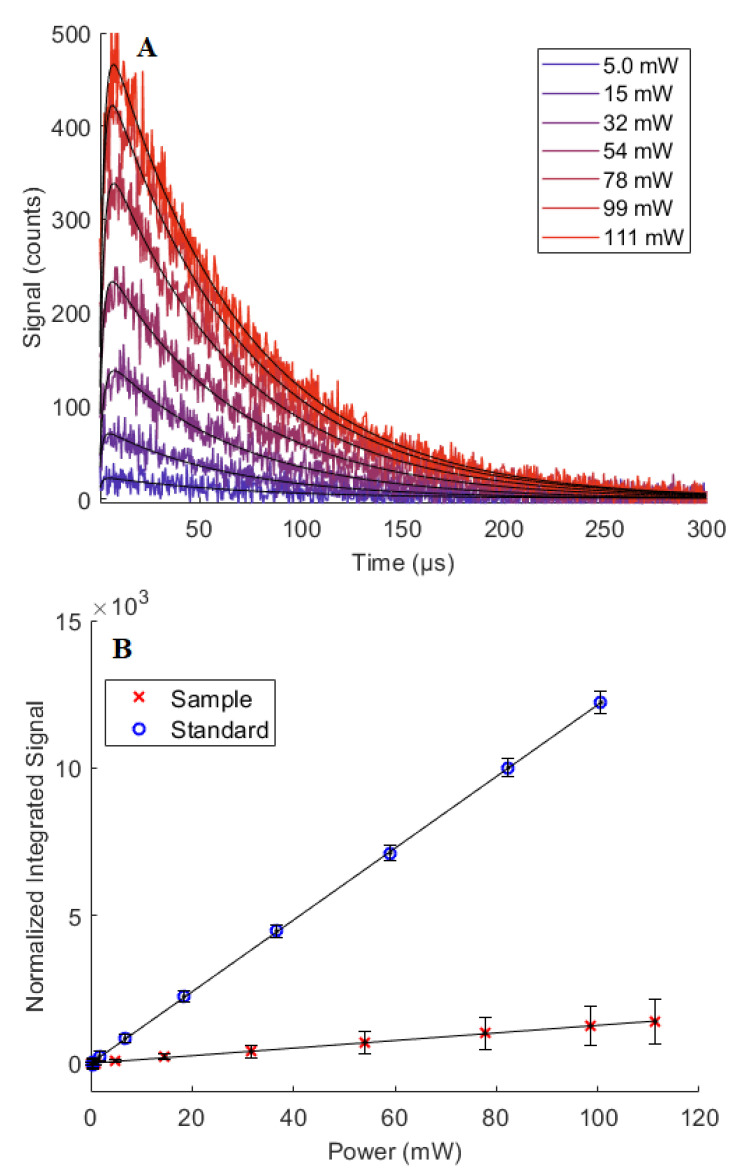
(**A**) Representative time-resolved O_2_(a^1^Δ_g_) → O_2_(X^3^Σ_g_^−^) phosphorescence traces recorded at 1275 nm upon 420 nm pulsed laser irradiation of Athens Green in air-saturated D_2_O at pD = 5.0. For the triplet state photosensitized production of O_2_(a^1^Δ_g_) in water, where the concentration of oxygen is comparatively low, it is common to discern a rise in the time-resolved signal corresponding to the comparatively slow rate of O_2_(a^1^Δ_g_) production. Such data are quantified using a difference of two exponential functions [27,39], and this fitting function is superimposed on each kinetic trace. (**B**) The integrated signal amplitude, normalized by the O_2_(a^1^Δ_g_) lifetime, *τ*_Δ_, and the sample absorbance, plotted as a function of the incident laser power. The slopes of the linear fits are proportional to *φ*_Δ_.

Quantum yields of O_2_(a^1^Δ_g_) production, *φ*_Δ_, were obtained in the standard way by comparing the intensity of the fluorescein-sensitized O_2_(a^1^Δ_g_) phosphorescence signal to the corresponding signal from O_2_(a^1^Δ_g_) produced by a reference sensitizer, using data obtained over a range of incident laser powers [40]. For our present experiments, the reference used was phenalen-1-one-2-sulfonic acid (PNS) [41]. Representative data for Athens Green are shown in Figure 6.

All our compounds sensitize the production of O_2_(a^1^Δ_g_) in readily detectable amounts. As expected from previous studies [12,14], values of *φ*_Δ_ are small under alkaline conditions where the fluorescein dianion predominates (Table 3). This is consistent with the large quantum yields of fluorescence and oxygen-independent fluorescence lifetimes observed under the same conditions (vide supra). Saturating the solution with oxygen does not significantly increase *φ*_Δ_ indicating that most of the expected precursor to O_2_(a^1^Δ_g_), the fluorescein triplet state, is quenched by oxygen under air-saturated conditions (Table 3).

**Figure 7:**
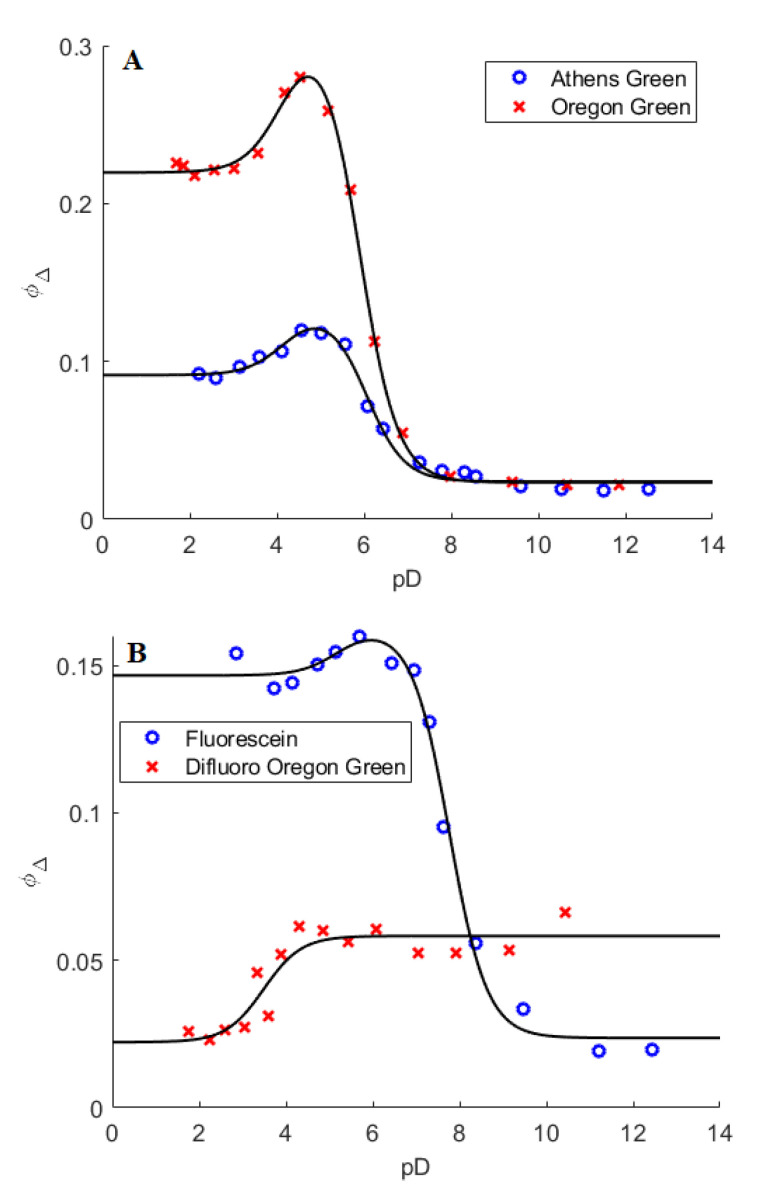
The O_2_(a^1^Δ_g_) quantum yield, *φ*_∆_, plotted as a function of the pD in air-saturated D_2_O-based solutions for (**A**) Athens Green and Oregon Green, and (**B**) fluorescein and difluoro Oregon Green. The solid lines are numerical fits to Equation (1). Differences in p*K*a and *φ*_∆_ values are apparent in these plots, and the pertinent numbers are shown in Table 4. For a given data point, the error is approximately 10% of the value shown.

Upon acidification of fluorescein and both difluorofluoresceins, *φ*_Δ_ increases to a maximum value at a pD where the monoanion dominates the equilibria (Figure 7). Thereafter, further acidification results in a slight decrease in *φ*_Δ_. Most importantly, this pD-dependent increase in the O_2_(a^1^Δ_g_) yield is comparatively small for Athens Green (from ~0.02 to 0.12 in an air-saturated solution), whereas it is much larger for Oregon Green (from ~0.02 to 0.28, likewise in an air-saturated solution).

Although pD dependent changes in *φ*_Δ_ for difluoro Oregon Green are very small (Table 3) and are arguably smaller than our absolute accuracy in determining *φ*_Δ_, the relative changes obtained upon systematically decrementing the pD show a clear decrease in *φ*_Δ_ upon acidification to form the mono anion (Figure 7B). As such, the pD dependent behavior of this tetrafluorinated fluorescein is distinctly different from that of the difluoro and fluorine-free analogs with respect to O_2_(a^1^Δ_g_) production. Based on the data that we currently have available, we are not able to explain this observation. Moreover, we are likewise currently hesitant to speculate on the origins of the more pronounced difference in *φ*_Δ_ between the non-symmetrical and symmetrical difluorofluoresceins. In themselves, these observations are fodder for a detailed independent study.

At any given pD, the measured value of the O_2_(a^1^Δ_g_) quantum yield, *φ*_∆_^meas^, will reflect contributions from the dianionic (da), the monoanionic (ma), and the neutral (n) species, and we can model the pD-dependence of *φ*_∆_^meas^ shown in Figure 7 with the function in Equation (1).

(1) $$\varphi_{\Delta }^{\mathrm{meas}}=\varphi_{\Delta }^{\mathrm{da}}+\;\frac{\varphi_{\Delta }^{\mathrm{ma}}-\varphi_{\Delta }^{\mathrm{da}}}{1+10^{\mathrm{pD}-pK_{a1}^{\;}}}+\frac{\varphi_{\Delta }^{n}-\varphi_{\Delta }^{\mathrm{ma}}}{1+10^{\mathrm{pD}-pK_{a2}^{\;}}}$$

In this treatment, *φ*_∆_^da^, *φ*_∆_^ma^, and *φ*_∆_^n^ do not reflect the actual quantum yields of O_2_(a^1^Δ_g_) production because we do not account for pD-dependent changes in the absorbance of each species. Nevertheless, *φ*_∆_^meas^ represents the weighted average of relative quantum yields from the respective pD-dependent components. In Table 4, we show parameters obtained from the application of Equation (1) to our pD-dependent data shown in Figure 7. Note that p*K*_a_ values obtained from D_2_O-based solutions are not expected to be identical to those obtained from H_2_O-based solutions [30].

The results shown in Table 4, confirm that fluorination decreases the p*K*_a_ values of fluorescein. More importantly, the relative values of *φ*_∆_ obtained through Equation (1) indicate that under all conditions, non-symmetric Athens Green produces appreciably less O_2_(a^1^Δ_g_) than the symmetric analog, Oregon Green.

### 2.3. Rate Constants for the Fluorescein-Mediated Removal of O_2_(a^1^Δ_g_)

To complement pD-dependent measurements of O_2_(a^1^Δ_g_) production sensitized by the fluorescein derivatives, we set out to monitor pD-dependent rate constants for O_2_(a^1^Δ_g_) removal by these same derivatives. For this latter exercise, it is useful to quantify not just the rate constant for total removal, *k*_total_, but to perform experiments that distinguish the rate of removal via the fluorescein-mediated physical deactivation of O_2_(a^1^Δ_g_) to O_2_(X^3^Σ_g_^−^), *k*_phys_, from the rate of O_2_(a^1^Δ_g_) removal via a chemical reaction with the fluorescein, *k*_chem_. Although the photobleaching mechanisms of fluorescein derivatives depend on whether oxygen is present or not [3,5,6,7], the magnitudes of *k*_phys_ and *k*_chem_ are certainly relevant for systems in which O_2_(a^1^Δ_g_) is produced, either by the fluorescein derivative itself or by another chromophore in the system.

#### 2.3.1. Rate Constants for the Total Removal of O_2_(a^1^Δ_g_)

**Figure 8:**
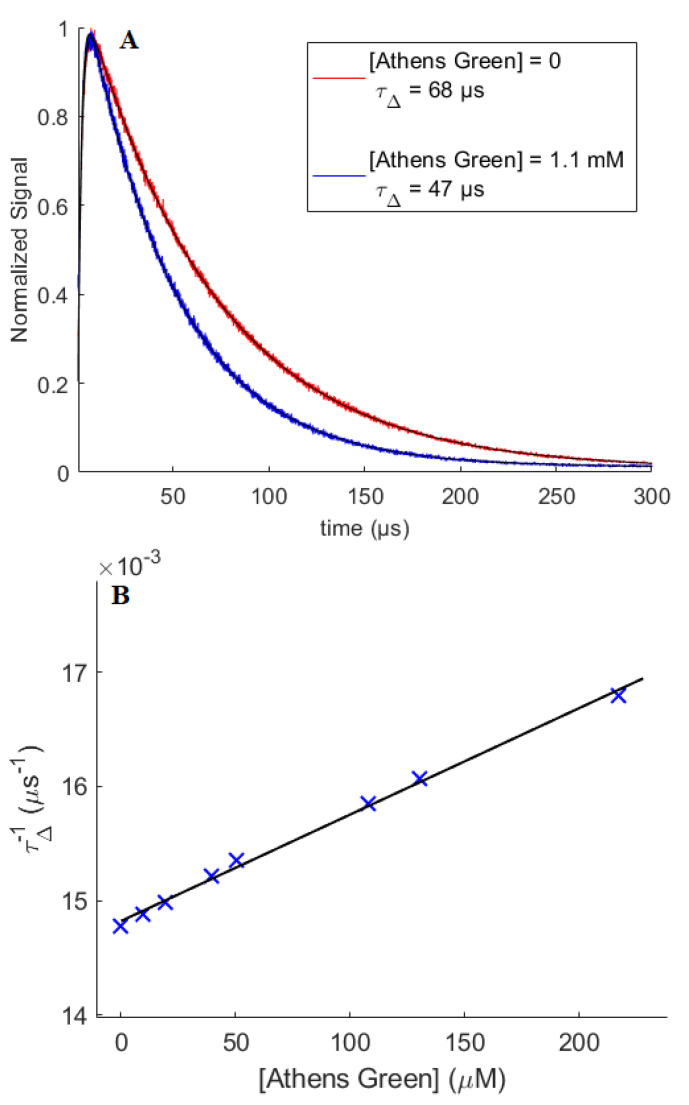
(**A**) Representative time-resolved 1275 nm O_2_(a^1^Δ_g_) phosphorescence traces recorded upon 670 nm pulsed laser irradiation of AlPcS_4_ in air-saturated D_2_O-PBS at pD = 7.8 in the presence of Athens Green, the concentration of which is shown in the legend. (**B**) The inverse lifetime of O_2_(a^1^Δ_g_), *τ*_Δ_^−1^, plotted as a function of the concentration of Athens Green, obtained from experiments such as those shown inFigure 8A. The solid black line is a linear fit to the data, the slope of which yields *k*_total_. The size of the symbols in Figure 8B represent the errors on each data point.

The rate constant for total solute-mediated O_2_(a^1^Δ_g_) removal, *k*_total_, which is the sum of *k*_phys_ and *k*_chem_, is readily obtained from time-resolved O_2_(a^1^Δ_g_) phosphorescence measurements performed as a function of the solute concentration. This experiment is best performed using a method for O_2_(a^1^Δ_g_) production that is independent of the solute used to mediate O_2_(a^1^Δ_g_) removal. To this end, we used Al(III) phthalocyanine tetrasulfonic acid, AlPcS_4_, as an O_2_(a^1^Δ_g_) photosensitizer [42]. Like its disulfonated analog, the AlPcS_4_-sensitized yield of O_2_(a^1^Δ_g_) will likely depend on pH [43]. For our kinetic experiments, however, this parameter is irrelevant as long as the yield is sufficiently large to yield good O_2_(a^1^Δ_g_) phosphorescence signals. Most importantly, the absorption spectrum of AlPcS_4_ is sufficiently red-shifted relative to those of our fluorescein derivatives that, upon irradiation, we avoid exciting the fluorescein itself [44] (also see Supplementary Materials). Representative data from these experiments are shown in Figure 8, and the values of *k*_total_ thus obtained are shown in Table 5.

#### 2.3.2. Rate Constants for the Removal of O_2_(a^1^Δ_g_) by Reaction

**Figure 9:**
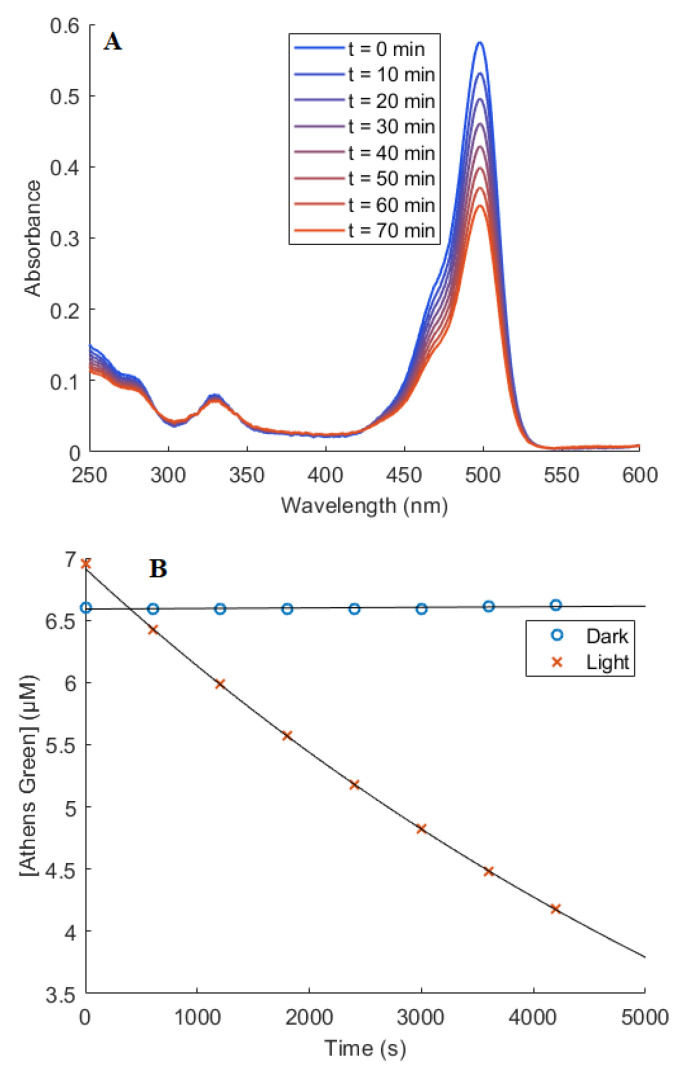
(**A**) Absorption spectra of Athens Green in D_2_O-PBS (pD 7.8) recorded as a function of the elapsed irradiation time of the O_2_(a^1^Δ_g_) sensitizer AlPcS_4_ at 670 nm. The absorption spectrum of AlPcS_4_ does not change under these conditions and was subtracted to yield the data shown. (**B**) The concentration of Athens Green as a function of AlPcS_4_ irradiation time (crosses). An independent sample, kept in the dark and monitored at identical intervals, did not show detectable absorption changes. The solid lines are fits to the pertinent kinetic rate equations for reactions with O_2_(a^1^Δ_g_) [45]. The size of the symbols in Figure 9B represent the errors on each data point.

The magnitude of *k*_chem_ can be independently determined using experiments in which O_2_(a^1^Δ_g_)-mediated changes in the fluorescein absorption spectra are monitored. The general kinetic approach we used is described elsewhere [45], and representative examples of our data are shown in Figure 9. For these experiments, we again produced O_2_(a^1^Δ_g_) upon irradiation of AlPcS_4_ at 670 nm. We ascertained that, over the period in which appreciable changes in the fluorescein spectra were observed, we saw no change in the AlPcS_4_ spectrum. Values of *k*_chem_ thus obtained are shown in Table 5.

#### 2.3.3. Interpreting Relative Changes in the Removal Rate Constants

We first note that, with the change in pD from 7.8 to 5 and the associated protonation of the dianion to yield the monoanion, all of the rate constants for O_2_(a^1^Δ_g_) removal decrease for both Oregon Green and Athens Green (Table 5). This is arguably expected given that O_2_(a^1^Δ_g_) is an electrophile, and it is consistent with the general observation that electron-rich molecules are most efficient at removing/deactivating O_2_(a^1^Δ_g_) [46]. Moreover, our data indicate that Oregon Green and Athens Green both predominantly remove O_2_(a^1^Δ_g_) via physical deactivation to O_2_(X^3^Σ_g_^−^) as opposed to a chemical reaction (i.e., *k*_phys_/*k*_chem_ > 2; Table 5). Beyond these similarities, however, and as outlined below, there are clear differences in the rate constants for O_2_(a^1^Δ_g_) removal by Oregon Green and Athens Green, as there were with the yields of photosensitized O_2_(a^1^Δ_g_) production (Table 3).

Overall, and irrespective of the pD, Athens Green is a poorer quencher of O_2_(a^1^Δ_g_) than Oregon Green (i.e., *k*_total_(Athens Green) < *k*_total_(Oregon Green)). This difference in *k*_total_ principally reflects the respective values of *k*_phys_ (Table 5). Moreover, of all the fluorescein derivatives we examined, the absolute magnitude of *k*_phys_ is the largest for Oregon Green. In turn, this results in a *k*_phys_/*k*_chem_ ratio for Oregon Green that is over 10 times greater than that for Athens Green. Of the postulated mechanisms for solute-mediated O_2_(a^1^Δ_g_) deactivation to O_2_(X^3^Σ_g_^−^), processes that occur with rate constants of ~10^6^–10^7^ s^−1^ M^−1^, as we have for these fluoresceins, focus on the role played by the oxygen-quencher charge-transfer (CT) state [47,48,49,50]. The thesis is that, for a compound M that can better donate charge to oxygen, the M^+.^O_2_^−.^ CT state will play a greater role in facilitating the O_2_(a^1^Δ_g_) → O_2_(X^3^Σ_g_^−^) transition. Thus, one hypothesis to account for our data is that the M-O_2_ CT state for Oregon Green plays a greater role than the corresponding CT state for Athens Green in the process of deactivating O_2_(a^1^Δ_g_).

Organic dye molecules and fluorophores, including the fluoresceins, photobleach by a variety of mechanisms in oxygen-containing systems [4,5,6,7]. These photobleaching reactions are undesired in many microscopy applications where a stable fluorophore is an attribute [51]. This is also true for organic solar cells where a stable light-absorbing compound is desired [52]. Reactions with O_2_(a^1^Δ_g_) constitute one important channel for such photodegradation. With this in mind, the magnitude of *k*_chem_ becomes important. Our data indicate that, at pD 7.8, the rate constant for the reaction of O_2_(a^1^Δ_g_) with Athens Green is approximately three times greater than the corresponding rate constant for the reaction of O_2_(a^1^Δ_g_) with Oregon Green (Table 5). At pD 5, although the difference in *k*_chem_ for these two molecules becomes negligible, a pronounced dark reaction contributes to the bleaching of Oregon Green whereas Athens Green is stable in the dark (see Supplementary Materials). An attempt to interpret these observations must include a detailed study of the reaction products and this, in itself, will be a challenging independent study. Although the judicious addition of substituent groups to Athens Green could reduce reactivity with O_2_(a^1^Δ_g_), the absolute value of *k*_chem_ is already small enough that such reactions will not preclude the use of Athens Green as a fluorescent probe in many applications. This latter point is reinforced by the fact that the yield of O_2_(a^1^Δ_g_) produced upon irradiation of Athens Green is less than that of Oregon Green (Table 3).

Thus, our data clearly show that, if a benign fluorescence probe is desired, Athens Green is a better choice than Oregon Green; Oregon Green will initiate and interfere with an O_2_(a^1^Δ_g_)-mediated process to a greater extent than Athens Green.

## 3. Conclusions

We synthesized a non-symmetrical difluorinated derivative of fluorescein, calling this compound Athens Green. Its symmetrical difluorinated complement is the well-established compound called Oregon Green. We ascertained that the oxygen-dependent photophysics and photochemistry of Athens Green differ appreciably from that of Oregon Green. In particular, Athens Green produces less O_2_(a^1^Δ_g_) upon irradiation and does not remove O_2_(a^1^Δ_g_) as efficiently as Oregon Green. As such, Athens Green would arguably be a more benign fluorescent probe in photosystems that involve O_2_(a^1^Δ_g_). On a more general level, our results show that the site of fluorination in a given molecule can have a profound effect on photophysical properties pertinent to the use of that molecule as a sensor. Thus, given the advantages of using a fluorinated fluorescein derivative over using fluorescein itself (i.e., the fluorinated compound can be used over a wider pH range), and given that fluorescein itself has already been established as a useful probe in a wide range of disciplines [1], Athens Green should be a welcome addition to the toolbox of fluorescent probes.

## 4. Materials and Methods

### 4.1. Synthesis of 2-(2,4-dihydroxybenzoyl)benzoic Acid (**1**)

Prepared as described in the literature [53]. Aluminum trichloride (1.08 g, 8.1 mmol) was added to a solution of **PA** (400 mg, 2.7 mmol) and **R** (273 mg, 2.7 mmol) in dry nitrobenzene (6 mL) under argon, and the mixture was degassed (Ar bubbling) for 30 min. The mixture was stirred for 24 h, decanted into a vigorously stirred 1:1 biphasic mixture of 0.5 N HCl (aq)/hexane (30 mL), the obtained mixture was stirred for 2 h, and the precipitate was filtered and washed with water and hexane. The resulting impure product was crystallized from a methanol/water mixture to afford **1** as an off-white powder (564 mg, 81%): ^1^H-NMR (200 MHz, DMSO-d_6_) δ 13.22 (bs, 1H, COOH), 12.25 (s, 1H, OH), 10.75 (s, 1H, OH), 8.04–7.96 (m, 1H, H-6), 7.75–7.59 (m, 2H, H-4, H-5), 7.42 (dd, *J* = 7.1, 1.4 Hz, 1H, H-3), 6.92 (d, *J* = 8.6 Hz, 1H, H-6′), 6.35–6.25 (m, 2H, H-3′, H-5′). ^13^C NMR (50 MHz, DMSO-d_6_) δ 200.54, 166.76, 164.93, 164.17, 140.08, 134.84, 132.43, 130.07, 129.81, 129.42, 127.52, 113.33, 108.34, 102.51. ES-HRMS m/z for C_14_H_7_F_2_O_5_ [M-H]^-^: calcd. 293.03, found 293.03.

### 4.2. Synthesis of 2-(3,5-difluoro-2,4-dihydroxybenzoyl)benzoic Acid (**2**)

Aluminum trichloride (1.08 g, 8.1 mmol) was added in a solution of **PA** (400 mg, 2.7 mmol) and 2,4-difluororesorcinol (395 mg, 2.7 mmol) in dry nitrobenzene (10 mL) under argon, and the resulting mixture was degassed (Ar bubbling) for 30 min. The mixture was stirred at 120 °C for 24 h, decanted into a vigorously stirred 1:1 biphasic mixture of 1 N HCl (aq)/hexane (40 mL), the mixture was stirred for 2 h, and the precipitate was filtered and washed with water and hexane. The resulting impure product was crystallized from a methanol/water mixture to afford **2** as an off-white powder (420 mg, 53%): ^1^H-NMR (200 MHz, CD_3_OD) δ 8.11 (app d, *J* = 7.2 Hz, 1H, H-6), 7.77–7.59 (m, 2H, H-4, H-5), 7.39 (app d, *J* = 7.1 Hz, 1H, H-3), 6.55 (app d, *J* = 11.0, 1H, H-6′). ^19^F-NMR (188 MHz, CD_3_OD) δ -146.43 (dd, *J* = 11.0, 6.3 Hz, 1F, F-5′), -160.65 (app d, *J* = 6.3 Hz, 1F, F-6′). ^13^C-NMR (75 MHz, CD_3_OD) δ 202.77, 168.47, 150.17 (dd, *J* = 12.6, 2.3 Hz), 145.87 (dd, *J* = 236.2, 4.3 Hz), 143.16 (dd, *J* = 17.9, 12.8 Hz), 142.02 (dd, *J* = 241.5, 5.4 Hz), 141.20, 133.66, 131.56, 131.14, 130.61, 128.40, 113.33 (dd, *J* = 20.6, 2.9 Hz), 112.74 (dd, *J* = 6.7, 2.3 Hz). ES-HRMS m/z for C_14_H_7_F_2_O_5_ [M-H]^-^: calcd. 293.0262, found 293.0239.

### 4.3. Synthesis of 2-(5,7- Difluoro-6-hydroxy-3-oxo-3h-xanthen-9-yl)benzoic Acid (Athens Green, **3**)

A mixture of **2** (200 mg, 0.68 mmol) and **R** (69 mg, 0.68 mmol) in methanesulfonic acid (3 mL) was stirred under argon for 4 h. The mixture was decanted into crushed ice (60 mL), and the resulting mixture was brought to room temperature and extracted with ethyl acetate (3 × 20 mL). The combined organic phases were washed with water, dried and the solvent was evaporated. The residue was suspended in water (10 mL), basified with triethylamine (pH≈10), and the resulting dark solution was refluxed for 10 min and left to cool down. The mixture was acidified with 1N HCl (pH≈2), extracted with ethyl acetate (3 × 20 mL), the combined organic phases were washed with water, dried and the solvent was evaporated. The residue was subjected to column chromatography (EtOAc/petroleum ether 10–100%) yielding **3** (51 mg, 15%) as an orange powder. ^1^H-NMR (200 MHz, DMSO-d_6_) δ 10.51 (bs, 2H, OH), 8.01 (d, *J* = 7.3 Hz, 1H, H-6), 7.85–7.67 (m, 2H, H-4, H-5), 7.33 (d, *J* = 7.2 Hz, 1H, H-3), 6.76 (s, 1H, H-4′), 6.64–6.55 (m, 1H, H-1′, H-2′), 6.40 (dd, *J* = 10.9, 2.2 Hz, 1H, H-8′). ^19^F-NMR (188 MHz, DMSO-d_6_) δ-136.93 (bs, 1F, F-5′, -154.06 (bs, 1F, F-7′). ^13^C-NMR (75 MHz, DMSO-d_6_) δ 168.78, 160.26, 151.96, 151.57, 148.83 (dd, *J* = 238.6 Hz), 140.93 (dd, *J* = 243.8, 6.6 Hz), 137.92 (d, *J* = 10.1 Hz), 137.02 (m), 136.04, 130.76, 129.50, 126.46, 125.50, 124.63, 113.85, 109.67 (d, *J* = 7.6 Hz), 109.45, 108.54 (dd, *J* = 20.9, 3.1 Hz), 102.79, 82.58. ES-HRMS m/z for C_20_H_9_F_2_O_5_ [M-H]^-^: calcd. 367.0418, found 367.0429.

### 4.4. Materials

Fluorescein was obtained from Sigma-Aldrich (>95%) and 2′,7′-difluorofluorescein (i.e., Oregon Green 488^TM^) was obtained from Thermo Fisher Scientific (>95%). The tetrafluoro substituted fluorescein derivative, 4′,5′-difluoro Oregon Green, was prepared using the procedure of Sun et al. [17]. Al(III) phthalocyanine tetrasulfonic acid (AlPcS_4_, >95%) was obtained from Frontier Scientific and Rhodamine 6G (R6G, >99%) from Sigma Aldrich, and both were used as received. The sulfonated derivative of 1H-phenalenone (PNS) was synthesized as described by Nonell et al. [54]. Heavy water (D_2_O, 99.9% D) was obtained from EurisoTop, whereas H_2_O was purified by filtration (Milli-Q system by Millipore Corporation).

H_2_O- and D_2_O-based phosphate-buffered saline (PBS) solutions were prepared using commercially available PBS tablets (Sigma-Aldrich). The pH of H_2_O-solutions was adjusted using hydrochloric acid (HCl) and sodium hydroxide (NaOH), whereas the pD of D_2_O-based solutions was adjusted with deuterochloric acid (DCl) and sodium deuteroxide (NaOD). The respective acids and bases were obtained from Sigma-Aldrich.

The tendency of fluorescein and Oregon Green to undergo excited-state proton transfer reactions has been documented [16,55]. If a suitable proton donor or acceptor (such as a phosphate buffer) is present, this can affect the photophysics. Hence, care must be exercised with the amount of any substance used to control the pH of our solutions. In all cases, we used the minimal amount of acid/base necessary to obtain a given pH, or we used the minimal amount of PBS (Total phosphate concentration = 0.01 M, [NaCl] = 0.137 M, [KCl] = 2.7 mM). In this regard, a phosphate concentration of 10 mM is low, compared to what is generally necessary to affect fluorescein singlet state photophysics [55]. Furthermore, control experiments using buffered and un-buffered solutions showed no difference in selected photophysical properties at identical pH values. Hence, we conclude that, under our conditions, the data are not sensitive to excited state reactions involving the buffer.

### 4.5. Methods

The spectroscopic instruments and procedures we use to (a) monitor excited states, (b) generate and detect O_2_(a^1^Δ_g_), and (c) follow the ensuing O_2_(a^1^Δ_g_)-induced chemistry have been previously described [45,56,57]. All reported quantum yields of O_2_(a^1^Δ_g_) production were measured using PNS in D_2_O-PBS as the standard (*φ*_Δ_ = 0.97 ± 0.06) [41]. Fluorescence quantum yields were determined using Rhodamine 6G in D_2_O-PBS (*φ*_fl_ = 0.98 ± 0.015) or H_2_O-PBS (*φ*_fl_ = 0.92 ± 0.02) as the standard [31,58]. The pH of D_2_O solutions, as measured with a pH-meter (Knick pH-matic), were corrected using the general formula pD = pH + 0.4 [29].

## Figures and Tables

**Table 1:** Extinction coefficients at the peak of the absorption band (ε_max_), the absorption (λ_max_^abs^), and fluorescence (λ_max_^fl^) band maxima, and fluorescence (*φ*_fl_) quantum yields, all recorded from air-saturated PBS buffered solutions at pH = 7.4 or pD = 7.8.

Compound	ε_max_ (M^−1^cm^−1^)	λ_max_^abs^ (nm)	λ_max_^fl^ (nm)	*φ*_fl_	
	D_2_O/H_2_O ^a^	D_2_O/H_2_O ^a^	D_2_O/H_2_O ^a^	H_2_O	D_2_O
Fluorescein ^b^	73000 ± 2900	490 ± 1	514 ± 1	0.92 ± 0.02	0.98 ± 0.02
Oregon Green	81000 ± 2700	490 ± 1	514 ± 1	Not measured	0.92 ± 0.03
Athens Green	82800 ± 3300	498 ± 1	523 ± 1	0.86 ± 0.02	0.91 ± 0.02
Difluoro Oregon Green	86700 ± 2900	507 ± 1	532 ± 1	Not measured	0.88 ± 0.02

^a^ The extinction coefficients and peak maxima are the same in both H_2_O and D_2_O-based solutions. ^b^ In a PBS solution at pH = 7.4 or pD = 7.8, some fraction of the fluorescein molecules exist as monoanions. For all other compounds, the p*K*_a_ values are sufficiently small that these data principally reflect the characteristics of the dianion). The extinction coefficient we obtain for fluorescein at this pH value is smaller than what has been reported at pH 9.0 (90,000 M^−1^cm^−1^) [17].

**Table 2:** Lifetimes of the fluorescent state, *τ*_fl_, in H_2_O-PBS and D_2_O-PBS as a function of the concentration of dissolved oxygen.

Compound	*τ*_fl_ (ns) ^a^					
	H_2_O-PBS			D_2_O-PBS		
	N_2_	Air	O_2_	N_2_	Air	O_2_
Fluorescein	4.28	4.26	4.25	4.75	4.69	4.60
Oregon Green	4.30	4.30	4.23	4.52	4.53	4.40
Athens Green	4.46	4.34	4.30	4.94	4.87	4.74
Difluoro Oregon Green	4.06	4.00	3.90	4.91	4.84	4.76

^a.^Errors on all numbers are ±0.05 ns.

**Table 3:** Singlet oxygen quantum yields, *φ*_Δ_, recorded from air- and oxygen-saturated D_2_O-PBS solutions.

	*φ*_Δ_^a^			
Compound	pD = 7.8		pD = 5	
	Air-Saturated	O_2_-Saturated	Air-Saturated	O_2_-Saturated
Fluorescein ^b^	0.04	0.05	0.16 ^c^	0.19 ^c^
Oregon Green	0.02	0.03	0.28	0.34
Athens Green	0.02	0.04	0.12	0.14
Difluoro Oregon Green	0.05	0.07	0.06	0.06

^a^ Errors on all numbers are ±0.01. ^b^ At pD = 7.8, a small fraction of the fluorescein molecules exist in monoanionic form. For all other compounds, the p*K*a is sufficiently low that the data principally reflect the effects of the dianion (see Table 4). ^c^ Measured at pD = 6 (i.e., the maximum of the φ_Δ_ versus pD plot; see Figure 7).

**Table 4:** Relative O_2_(a^1^Δ_g_) quantum yields, *φ*_∆_, in air-saturated D_2_O for the neutral (n), monoanionic (ma), and dianionic (da) species, and the corresponding p*K*_a_ values for the interconversion between them, as obtained through Equation (1). ^a.^

Compound	*φ*_Δ_^da^	p*K*_a1_	*φ*_Δ_^ma^	p*K*_a2_	*φ*_Δ_^n^
Fluorescein	0.03 ± 0.01	7.1 ± 0.3	0.17 ± 0.01	4.2 ± 0.2	0.15 ± 0.01
Oregon Green	0.02 ± 0.01	5.4 ± 0.2	0.34 ± 0.02	3.7 ± 0.2	0.24 ± 0.02
Athens Green	0.01 ± 0.01	5.7 ± 0.2	0.08 ± 0.01	3.4 ± 0.2	0.05 ± 0.01
Difluoro Oregon Green	0.04 ± 0.01	4.1 ± 0.2	0.03 ± 0.01	<2 ^b^	-^b^

^a.^The numbers shown here were obtained from fits of Equation (1) to plots of the singlet oxygen yield against pD (e.g., Figure 7). Recall also the expected difference of ~0.4 units in p*K*a values for experiments performed in H_2_O and D_2_O [30]. ^b.^The second p*K*a value of difluoro Oregon Green falls below our investigated pD range.

**Table 5:** Rate constants for the interaction with O_2_(a^1^Δ_g_) measured at two different pD values in air-saturated solutions.

Compound	pD = 7.8				pD = 5			
	*k*_total_ 10^6^ M^−1^s^−1^	*k*_chem_ 10^6^ M^−1^s^−1^	*k*_phys_^a^ 10^6^ M^−1^s^−1^	$\frac{k_{phys}}{k_{chem}}$	*k*_total_ 10^6^ M^−1^s^−1^	*k*_chem_ 10^6^ M^−1^s^−1^	*k*_phys_^a^ 10^6^ M^−1^s^−1^	$\frac{k_{phys}}{k_{chem}}$
Fluorescein	8.5 ± 0.8	2.0 ± 0.3	6.5 ± 1.1	3.3	4 ^b^	0.2 ^b^	3.8 ^b^	19 ^b^
Oregon Green	19 ± 2	0.6 ± 0.1	18.4 ± 3.2	31	9.1 ± 0.9	0.39 ± 0.09 ^c^	8.7 ± 2.0	22
Athens Green	6.0 ± 0.6	1.7 ± 0.2	4.3 ± 0.7	2.5	3.3 ± 0.3 ^d^	0.27 ± 0.06	3.0 ± 0.7	11
Difluoro OG ^e^	3.6 ± 0.4	0.8 ± 0.1	2.8 ± 0.5	3.5	-	-	-	-

^a^ Obtained from *k*_total_–*k*_chem_. Given the errors on *k*_chem_ and *k*_phys_, the error on the ratio of *k*_phys_/*k*_chem_ is sufficiently large (~20–25% of the value shown) that we choose to only conclude that physical deactivation of O_2_(a^1^Δ_g_) is more probable than chemical reaction with O_2_(a^1^Δ_g_) for these molecules (see text). ^b^ It was difficult to dissolve a sufficient amount of fluorescein to yield a non-negligible change in the O_2_(a^1^Δ_g_) lifetime when determining *k*_total_. For *k*_chem_, we accounted for a non-negligible change in the dark reaction. Thus, the errors on these numbers are large. As with the O_2_(a^1^Δ_g_) experiments, these experiments were also performed at pD 6. ^c^ There was a non-negligible dark reaction, and this change was subtracted from the change upon irradiation (see Supplementary Materials). ^d^ With 1% DMSO by volume. This was required to dissolve enough Athens Green to get an appreciable change in the O_2_(a^1^Δ_g_) lifetime. ^e^ Difluoro Oregon Green.

## Supplementary Materials

The following are available online at https://www.mdpi.com/1424-8220/20/18/5172/s1: NMR spectra, pD-dependent absorption and fluorescence spectra for difluoro Oregon Green, absorption spectra of AlPcS_4_, and plots used to determine *k*_chem_.

## References

[1] Lakowicz J.R. (2006). Principles of Fluorescence Spectroscopy.

[2] Robertson T.A., Bunel F., Roberts M.S. (2013). Fluorescein derivatives in intravital fluoresence imaging. Cells.

[3] Widengren J., Rigler R. (1996). Mechanisms of photobleaching investgated by fluorescence correlation spectroscopy. Bioimaging.

[4] Song L., Hennik E.J., Young I.T., Tanke H.J. (1995). Photobleaching kinetics of fluorescein in quantitative fluorescence microscopy. Biophys. J..

[5] Song L., Varma C.A.G.O., Verhoeven J.W., Tanke H.J. (1996). Influence of the triplet excited state on the photobleaching kinetics of fluorescein in microscopy. Biophys. J..

[6] Zheng Q., Jockusch S., Zhou Z., Blanchard S.C. (2014). The contribution of reactive oxygen species to the photobleaching of organic fluorophores. Photochem. Photobiol..

[7] Silva E.F.F., Pimenta F.M., Pedersen B.W., Blaikie F.H., Bosio G.N., Breitenbach T., Westberg M., Bregnhøj M., Etzerodt M., Arnaut L.G. (2016). Intracellular singlet oxygen photosensitizers: On the road to solving the problems of sensitizer degradation, bleaching and relocalization. Integr. Biol..

[8] Valdes-Aguilera O., Neckers D.C. (1989). Aggregation phenomena in xanthene dyes. Acc. Chem. Res..

[9] Lavis L.D., Rutkoski T.J., Raines R.T. (2007). Tuning the pKa of fluorescein to optimize binding assays. Anal. Chem..

[10] Klonis N., Sawyer W.H. (1996). Spectral properties of the prototropic forms of fluorescein in aqueous solution. J. Fluoresc..

[11] Martin M.M., Lindqvist L. (1975). The pH dependence of fluorescein fluorescence. J. Lumin..

[12] Holmehave J., Pedersen S.K., Jensen H.H., Ogilby P.R. (2015). Aarhus Green: A tetrafluoro-substituted derivative of fluorescein. ARKIVOC.

[13] Han J., Burgess K. (2010). Fluorescent indicators for intracellular pH. Chem. Rev..

[14] Pedersen S.K., Holmehave J., Blaikie F.H., Gollmer A., Breitenbach T., Jensen H.H., Ogilby P.R. (2014). Aarhus Sensor Green: A fluorescent probe for singlet oxygen. J. Org. Chem..

[15] Mchedlov-Petrossyan N.O., Vodolazkaya N.A., Gurina Y.A., Sun W.-C., Gee K.R. (2010). Medium effects on the prototropic equilibria of fluorescein fluoro derivatives in true and organized solution. J. Phys. Chem. B.

[16] Orte A., Crovetto L., Talavera E.M., Boens N., Alvarez-Pez J. (2005). Absorption and emission study of 2′,7′-difluorofluorescein and its excited-state buffer-mediated proton exchange reactions. J. Phys. Chem. A.

[17] Sun W.-C., Gee K.R., Klaubert D.H., Haugland R.P. (1997). Synthesis of fluorinated fluoresceins. J. Org. Chem..

[18] Mottram L.F., Boonyarattanakalin S., Kovel R.E., Peterson B.R. (2006). The Pennsylvania green fluorophore: A hybrid of oregon green and tokyo green for the construction of hydrophobic and ph-insensitive molecular probes. Org. Lett..

[19] Renikuntla B.R., Rose H.C., Eldo J., Waggoner A.S., Armitage B.A. (2004). Improved photostability and fluorescence properties through polyfluorination of a cyanine dye. Org. Lett..

[20] Hammershøj P., Kumar E.K.P., Harris P., Andresen T.L., Clausen M.H. (2015). Facile large-scale sythesis of 5- and 6-carboxyfluoresceins: Application for the Preparation of new fluorescent dyes. Eur. J. Org. Chem..

[21] Burdette S.C., Frederickson C.J., Bu W., Lippard S.J. (2003). ZP4, an improved neuronal Zn^2+^ sensor of the zinpyr family. J. Am. Chem. Soc..

[22] Lukhtanov E.A., Vorobiev A.V. (2008). Mild synthesis of asymmetric 2′-carboxyethyl-substituted fluoresceins. J. Org. Chem..

[23] Urano Y., Kamiya M., Kanda K., Ueno T., Hirose K., Nagano T. (2005). Evolution of fluorescein as a platform for finely tunable fluorescence probes. J. Am. Chem. Soc..

[24] Li J., Yao S.-Q. (2009). “Singapore Green”: A new fluorescent dye for microarray and bioimaging applications. Org. Lett..

[25] Martinez-Peragon A., Miguel D., Orte A., Mota A.J., Ruedas-Rama M.J., Justicia J., Alvarez-Pez J.M., Cuerva J.M., Crovetto L. (2014). Rational design of a new fluorescent “on/off” xanthene dye for phosphate detection in live cells. Org. Biomol. Chem..

[26] Butler A.R., Capon B., Rees C.W. (1969). Electrophilic Aromatic Substitution. Organic Reaction Mechansims.

[27] Ogilby P.R. (2010). Singlet oxygen: There is indeed something new under the sun. Chem. Soc. Rev..

[28] Bregnhøj M., Westberg M., Minaev B.F., Ogilby P.R. (2017). Singlet oxygen photophysics in liquid solvents: Converging on a unified picture. Acc. Chem. Res..

[29] Covington A.K., Paabo M., Robinson R.A., Bates R.G. (1968). Use of the glass electrode in deuterium oxide and the relation between the standardized pD (pa_D_) scale and the operational pH in heavy water. Anal. Chem..

[30] Krezel A., Bal W. (2004). A formula for correlating p*K*a values determined in D_2_O and H_2_O. J. Inorg. Biochem..

[31] Magde D., Wong R., Seybold P.G. (2002). Fluorescence quantum yields and their relation to lifetimes of rhodamine 6G and fluorescein in nine solvents: Improved absolute standards for quantum yields. Photochem. Photobiol..

[32] Alvarez-Pez J.M., Ballesteros L., Talavera E.M., Yguerabide J. (2001). Fluorescein excited-state proton exchange reactions: Nanosecond emission kinetics and correlation with steady-state fluorescence intensity. J. Phys. Chem. A.

[33] Thorning F., Jensen F., Ogilby P.R. (2020). Modeling the effect of solvents on nonradiative singlet oxygen deactivation: Going beyond weak coupling in intermolecular electronic-to-vibrational energy transfer. J. Phys. Chem. B.

[34] Battino R., Rettich T.R., Tominaga T. (1983). The solubility of oxygen and ozone in liquids. J. Phys. Chem. Ref. Data.

[35] Ware W.R. (1962). Oxygen quenching of fluorescence in solution: An experimental study of the diffusion process. J. Phys. Chem..

[36] Montalti M., Credi A., Prodi L., Gandolfi M.T. (2006). Handbook of Photochemistry.

[37] Gollmer A., Arnbjerg J., Blaikie F.H., Pedersen B.W., Breitenbach T., Daasbjerg K., Glasius M., Ogilby P.R. (2011). Singlet oxygen sensor green^®^: Photochemical behavior in solution and in a mammalian cell. Photochem. Photobiol..

[38] Pimenta F.M., Jensen R.L., Breitenbach T., Etzerodt M., Ogilby P.R. (2013). Oxygen-dependent photochemistry and photophysics of “miniSOG”, a protein-encased flavin. Photochem. Photobiol..

[39] Snyder J.W., Skovsen E., Lambert J.D.C., Poulsen L., Ogilby P.R. (2006). Optical detection of singlet oxygen from single cells. Phys. Chem. Chem. Phys..

[40] Scurlock R.D., Mártire D.O., Ogilby P.R., Taylor V.L., Clough R.L. (1994). Quantum yield of photosensitized singlet oxygen (a^1^Δ_g_) production in solid polystyrene. Macromolecules.

[41] Marti C., Jürgens O., Cuenca O., Casals M., Nonell S. (1996). Aromatic ketones as standards for singlet molecular oxygen O_2_(^1^Δ_g_) photosensitization. Time-resolved photoacoustic and near-IR emission studies. J. Photochem. Photobiol. A. Chem..

[42] Keir W.F., Land E.J., MacLennan A.H., McGarvey D.J., Truscott T.G. (1987). Pulsed Radition studies of photodynamic sensitizers: The nature of DHE. Photochem. Photobiol..

[43] Ostler R.B., Scully A.D., Taylor A.G., Gould I.R., Smith T.A., Waite A., Phillips D. (2000). The effect of pH on the photophysics and photochemistry of di-sulphonated aluminum phthalocyanine. Photochem. Photobiol..

[44] Dhami S., de Mello A.J., Rumbles G., Bishop S.M., Phillips D., Beeby A. (1995). Phthalocyanine fluorescence at high concentration: Dimers or reabsorption effect?. Photochem. Photobiol..

[45] Bregnhøj M., Krægpøth M.V., Sørensen R.J., Westberg M., Ogilby P.R. (2016). Solvent and heavy-atom effects on the O_2_(X^3^Σ_g_^-^) - O_2_(b^1^Σ_g_^+^) absorption transition. J. Phys. Chem. A.

[46] Wilkinson F., Helman W.P., Ross A.B. (1995). Rate constants for the decay and reactions of the lowest electronically excited singlet state of molecular oxygen in solution. An expanded and revised compilation. J. Phys. Chem. Ref. Data.

[47] Schweitzer C., Schmidt R. (2003). Physical mechanisms of generation and deactivation of singlet oxygen. Chem. Rev..

[48] Clennan E.L., Noe L.J., Szneler E., Wen T. (1990). Hydrazines: New charge-transfer physical quenchers of singlet oxygen. J. Am. Chem. Soc..

[49] Paterson M.J., Christiansen O., Jensen F., Ogilby P.R. (2006). Overview of theoretical and computational methods applied to the oxygen-organic molecule photosystem. Photochem. Photobiol..

[50] Jensen P.-G., Arnbjerg J., Tolbod L.P., Toftegaard R., Ogilby P.R. (2009). Influence of an intermolecular charge-transfer state on excited-state relaxation dynamics: Solvent effect on the methylnaphthalene-oxygen system and its significance for singlet oxygen production. J. Phys. Chem. A.

[51] Pawley J.B. (2006). Handbook of Biological Confocal Microscopy.

[52] Cheng P., Zhan X. (2016). Stability of organic solar cells: Challenges and strategies. Chem. Soc. Rev..

[53] Smith G.A., Metcalfe J.C., Clarke S.D. (1993). The design and properties of a series of calcium indicators which shift from rhodamine-like to fluorescein-like fluorescence on binding calcium. J. Chem. Soc. Perkin Trans..

[54] Nonell S., Gonzalez M., Trull F.R. (1993). 1H-Phenalen-1-one-2-sulfonic acid: An extremely efficient singlet molecular oxygen sensitizer for aqueous media. Afinidad.

[55] Paredes J.M., Crovetto L., Rios R., Orte A., Alvarez-Pez J.M., Talavera E.M. (2009). Tuned lifetime, at the ensemble and single molecule level, of a xanthenic fluorescent dye by means of a buffer-mediated excited state proton exchange reaction. Phys. Chem. Chem. Phys..

[56] Westberg M., Bregnhøj M., Etzerodt M., Ogilby P.R. (2017). Temperature sensitive singlet oxygen photosensitization by LOV-derived fluorescent flavoproteins. J. Phys. Chem. B.

[57] Arnbjerg J., Johnsen M., Frederiksen P.K., Braslavsky S.E., Ogilby P.R. (2006). Two-photon photosensitized production of singlet oxygen: Optical and optoacoustic characterization of absolute two-photon absorption cross sections for standard sensitizers in different solvents. J. Phys. Chem. A.

[58] Brouwer A.M. (2011). Standards for photoluminescence quantum yield measurements in solution (IUPAC Technical Report). Pure Appl. Chem..

