# Supplementary material for: Oxygen- and pH-Dependent Photophysics of Fluorinated Fluorescein Derivatives: Non-Symmetrical vs. Symmetrical Fluorination
Source: Sensors (Basel). 2020 Sep 10;20(18):5172. doi: 10.3390/s20185172 (PMC7570907; doi:10.3390/s20185172)
Supplement: Supplementary file 1 [file sensors-20-05172-s001.pdf]

Supporting Information

Oxygen- and pH-Dependent Photophysics of Fluorinated Fluorescein Derivatives: Non-Symmetrical vs. Symmetrical Fluorination

Ciaran K. McLoughlin,[1] Eleni Kotroni,[2] Mikkel Bregnhøj,[1] Georgios Rotas, [2]* Georgios C. Vougioukalakis,[2]* and Peter R. Ogilby[1]*

[1]Department of Chemistry, Aarhus University, DK-8000 Aarhus, Denmark
[2]Department of Chemistry, National and Kapodistrian University of Athens, Athens, Greece

* To whom correspondence should be addressed:

P. R. Ogilby (progilby@chem.au.dk)

G. C. Vougioukalakis (vougiouk@chem.uoa.gr)

G. Rotas (rotasgiorgos@hotmail.com)

Table of Contents:

	Page
NMR Spectra ……………………………………………………………………	S3
pD-Dependent Absorption and Fluorescence Spectra for Difluoro Oregon Green	S6
Absorption Spectrum of $AlPcS_4$ ………………………………………………..	S7
Plots Used to Determine k_{chem} at pD 5 …………………………………………	S8
References ………………………………………………...................................	S9

NMR Spectra

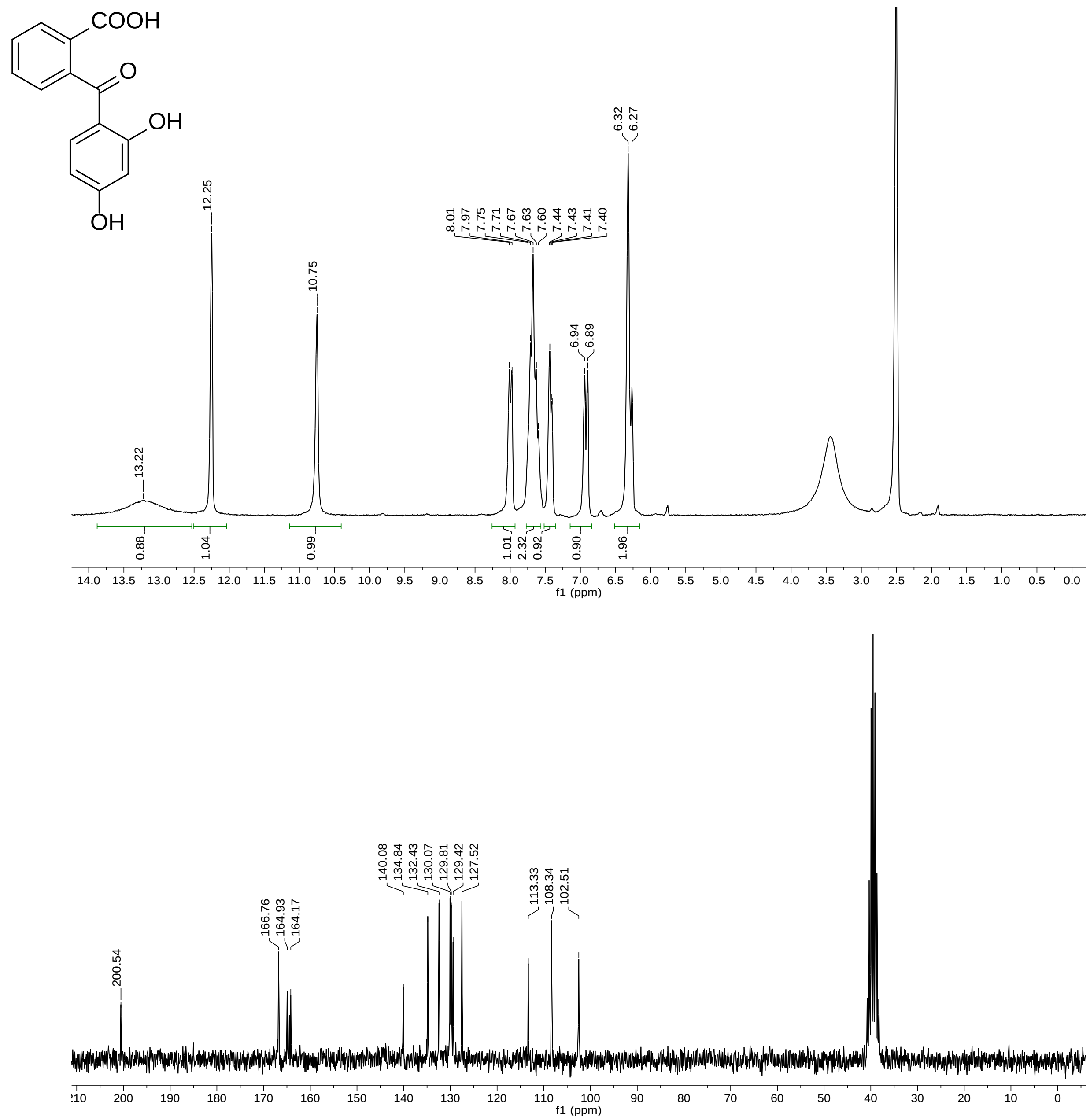

Figure S1. ^{1}H-NMR (200 MHz, top) and ^{13}C-NMR (50 MHz, bottom) of **1** in DMSO-d_6.

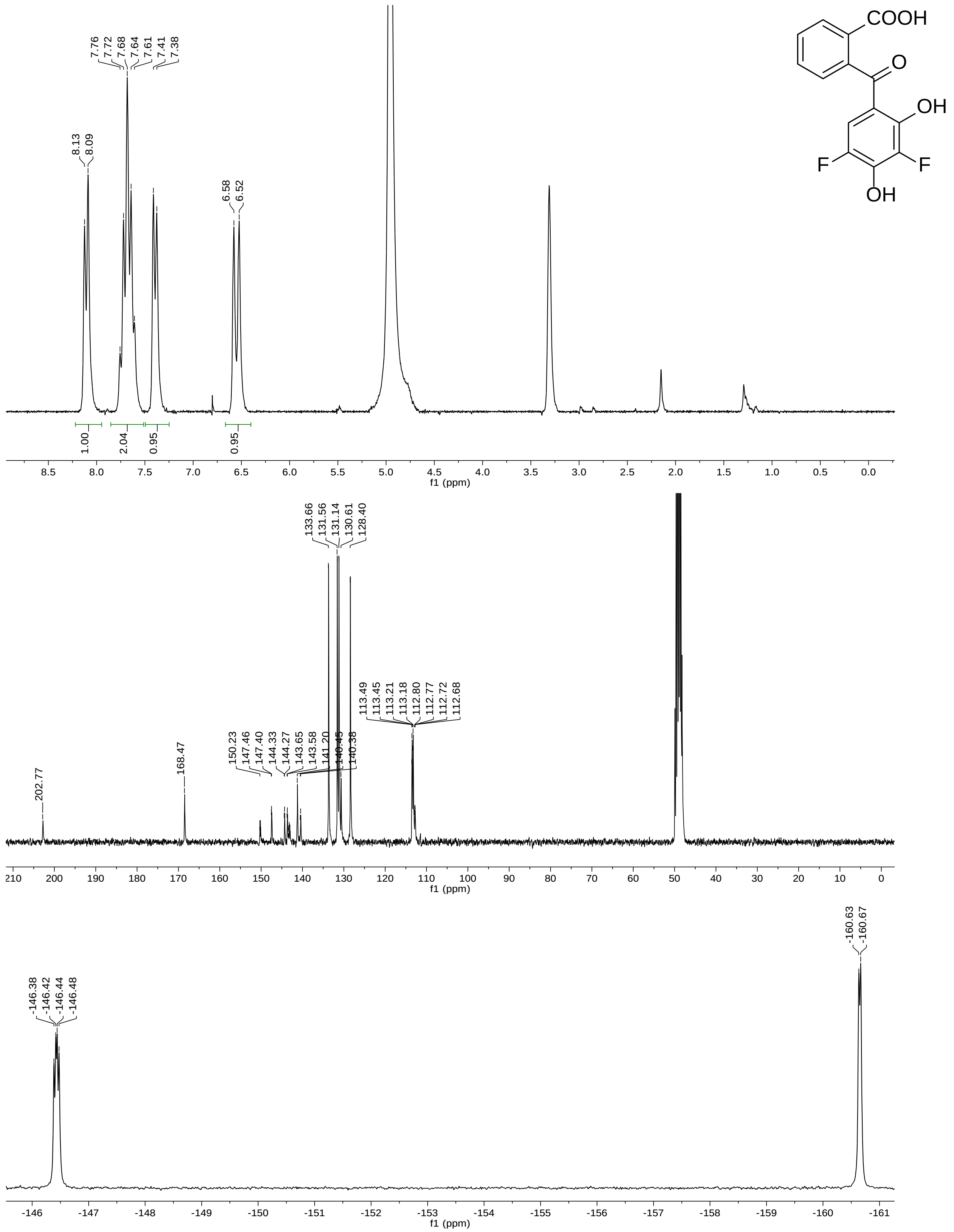

Figure S2. ^{1}H-NMR (200 MHz, top), ^{13}C-NMR (75 MHz, middle) and ^{19}F-NMR (188 MHz, bottom) of **2** in CD_3OD.

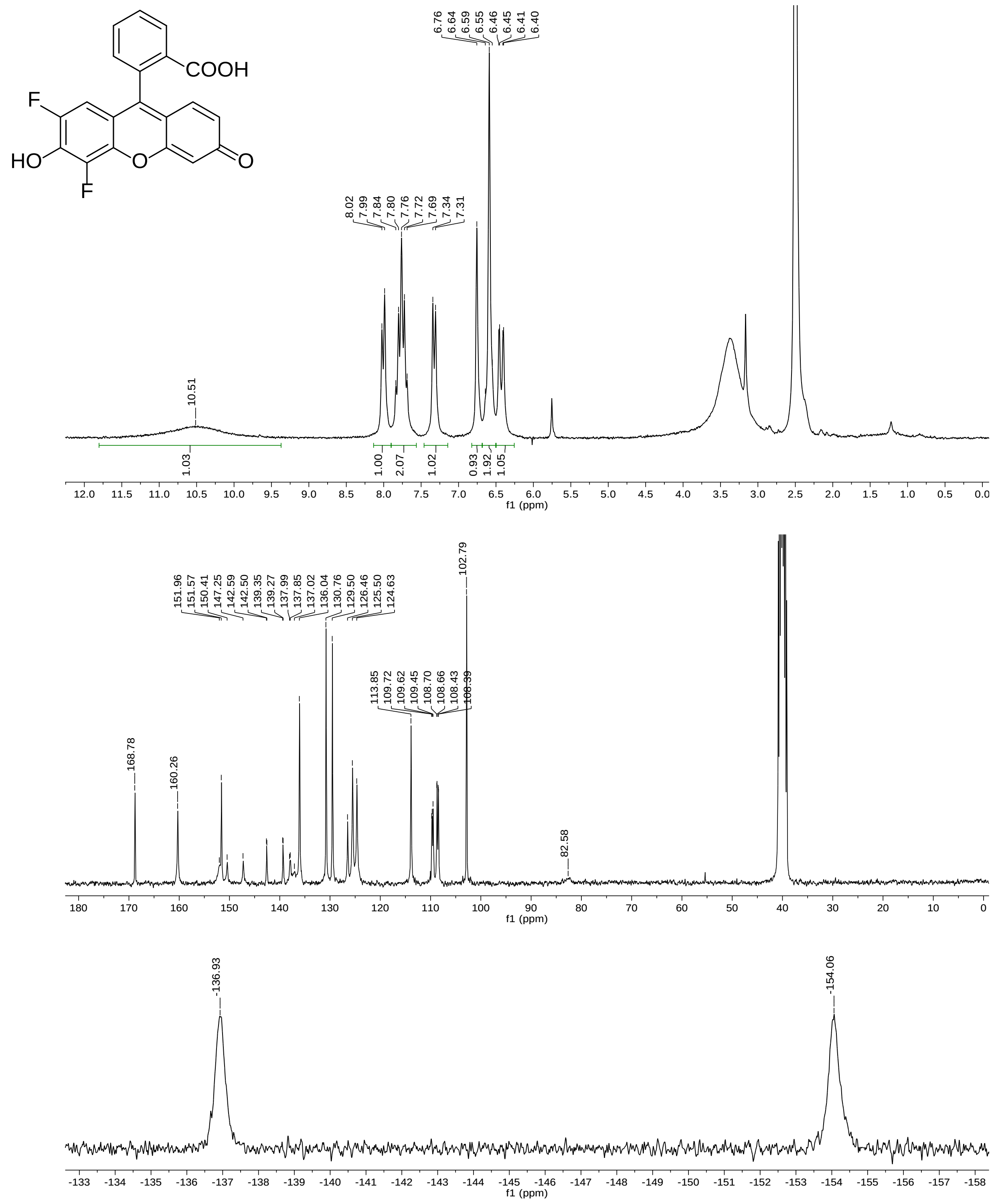

Figure S3. ^{1}H-NMR (200 MHz, top), ^{13}C-NMR (75 MHz, middle) and ^{19}F-NMR (188 MHz, bottom) of **3** in CD_3OD.

Absorption and Fluorescence Spectra for Difluoro Oregon Green

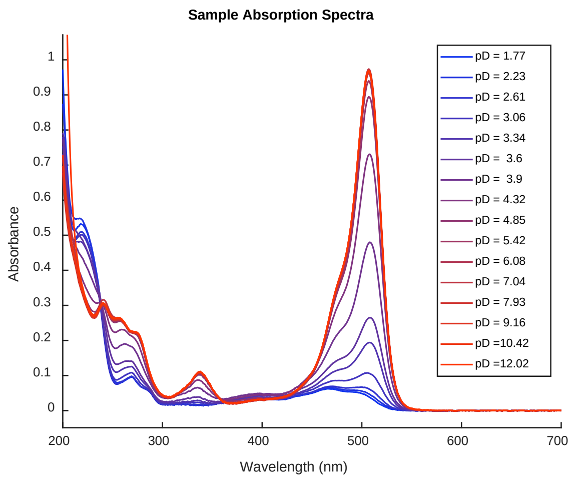

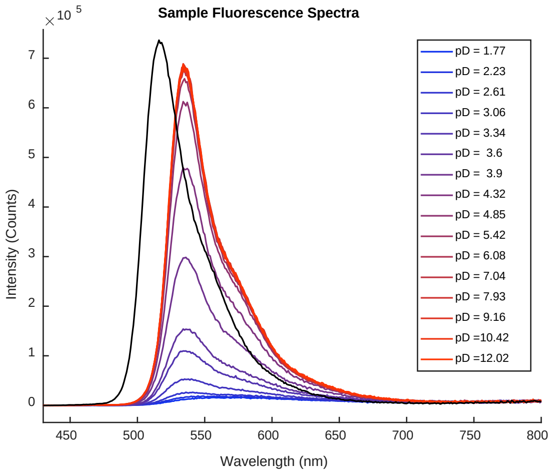

Figure S4. Absorption (top) and fluorescence (bottom) spectra of difluoro Oregon Green as a function of pD. The fluorescence spectra were normalized for pD dependent changes in the sample absorbance at the excitation wavelength of 420 nm. The fluorescence spectrum shown with a black line is that of fluorescein, which was used as a standard for the quantum yield studies.

Absorption Spectrum of $AlPcS_4$

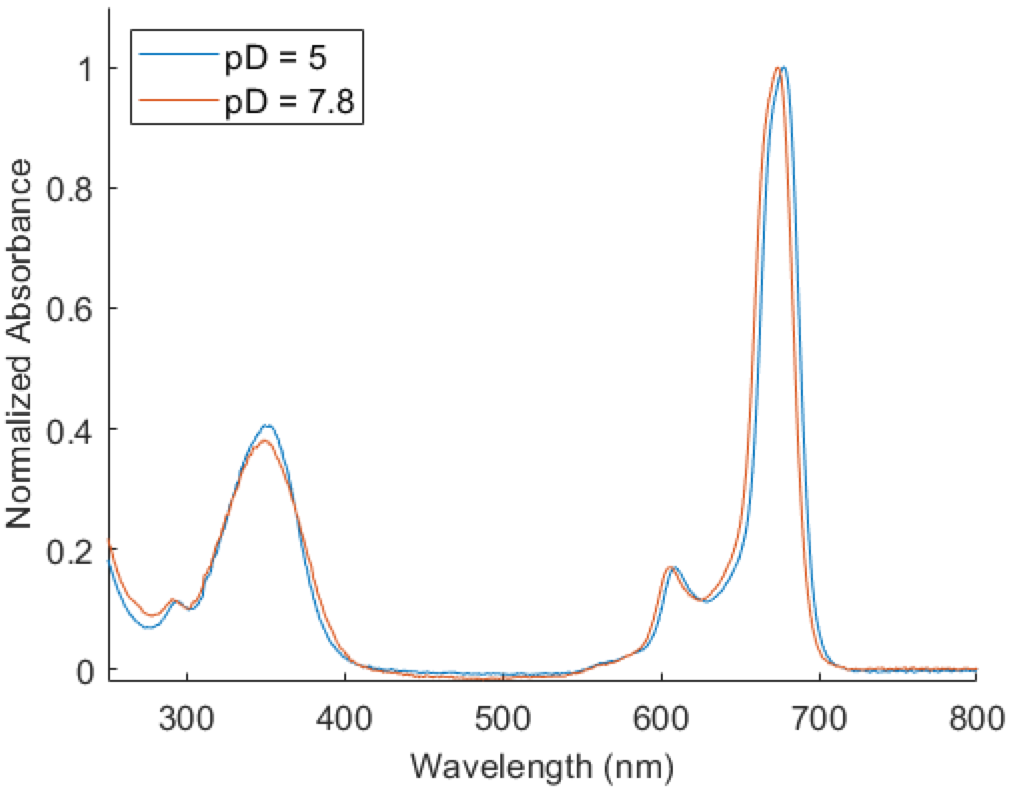

Figure S5. Absorption spectra of $AlPcS_4$ used as an independent $O_2(a^1\Delta_g)$ sensitizer in the experiments used to quantify rates of $O_2(a^1\Delta_g)$ removal by the fluorescein derivatives. Note that the absorbance of all fluorescein derivatives drops to 0 at ~ 550 nm (see Figure 4 in the main text).

Plots Used to Determine k_{chem} at pD 5

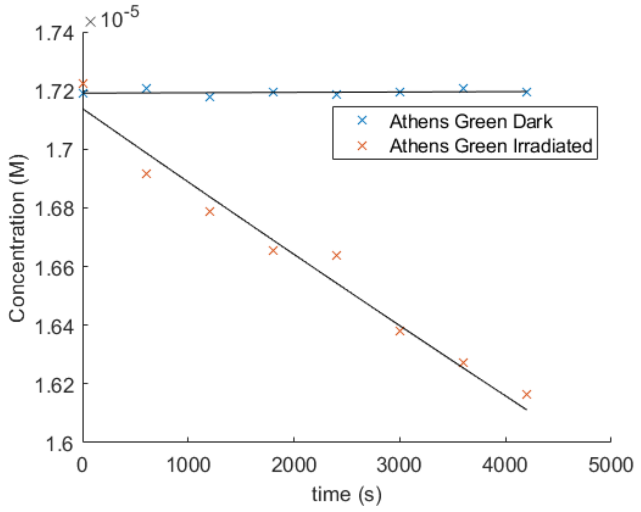

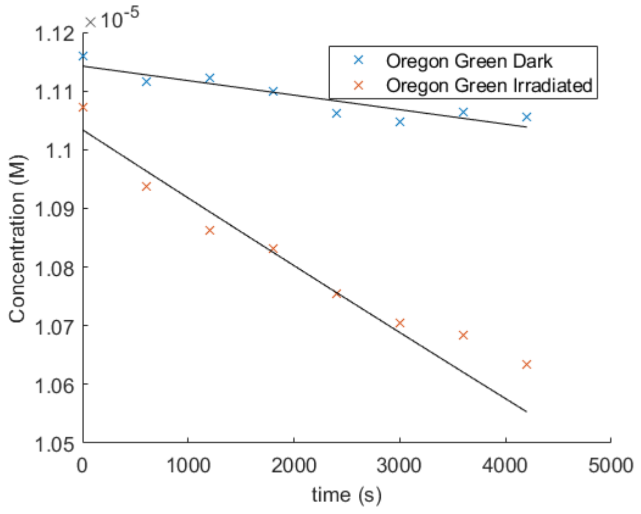

Figure S6. Plots of the concentration change of the specified fluorescein derivative against the elapsed irradiation time of the $O_2(a^1\Delta_g)$ sensitizer $AlPcS_4$ in solutions at pD 5. The solid lines reflect the general kinetic treatment published elsewhere for $O_2(a^1\Delta_g)$-mediated removal of a dissolved substrate [1]. Unique to these experiments is the fact that Oregon Green degrades in the dark. In all cases, to focus on the initial rate of removal, we excluded the last two data points from our fit (*i.e.*, cases where an appreciable amount of the fluorescein had been removed).

References

1. Bregnhøj, M.; Krægpøth, M. V.; Sørensen, R. J.; Westberg, M. and Ogilby, P. R. Solvent and Heavy-Atom Effects on the $O_2(X^3\Sigma_g^-)$ - $O_2(b^1\Sigma_g^+)$ Absorption Transition. *J. Phys. Chem. A* **2016**, *120*, 8285-8296.
